# A Cluster-Based Approach for Identifying Prognostic microRNA Signatures in Digestive System Cancers

**DOI:** 10.3390/ijms22041529

**Published:** 2021-02-03

**Authors:** Jun Zhou, Xiang Cui, Feifei Xiao, Guoshuai Cai

**Affiliations:** 1 Department of Environmental Health Sciences, Arnold School of Public Health, University of South Carolina, Columbia, SC 29208, USA; zhoujun.nmu@gmail.com (J.Z.); cuixiang2019@foxmail.com (X.C.); 2 Department of Epidemiology and Biostatistics, Arnold School of Public Health, University of South Carolina, Columbia, SC 29208, USA; XIAOF@mailbox.sc.edu

**Keywords:** miRNA, cluster analysis, digestive system cancers, prognostic marker

## Abstract

Cancer remains the second leading cause of death all over the world. Aberrant expression of miRNA has shown diagnostic and prognostic value in many kinds of cancer. This study aims to provide a novel strategy to identify reliable miRNA signatures and develop improved cancer prognostic models from reported cancer-associated miRNAs. We proposed a new cluster-based approach to identify distinct cluster(s) of cancers and corresponding miRNAs. Further, with samples from TCGA and other independent studies, we identified prognostic markers and validated their prognostic value in prediction models. We also performed KEGG pathway analysis to investigate the functions of miRNAs associated with the cancer cluster of interest. A distinct cluster with 28 cancers and 146 associated miRNAs was identified. This cluster was enriched by digestive system cancers. Further, we screened out 8 prognostic miRNA signatures for STAD, 5 for READ, 18 for PAAD, 24 for LIHC, 12 for ESCA and 18 for COAD. These identified miRNA signatures demonstrated strong abilities in discriminating the overall survival time between high-risk group and low-risk group (*p*-value < 0.05) in both TCGA training and test datasets, as well as four independent Gene Expression Omnibus (GEO) validation datasets. We also demonstrated that these cluster-based miRNA signatures are superior to signatures identified in single cancers for prognosis. Our study identified significant miRNA signatures with improved prognosis accuracy in digestive system cancers. It also provides a novel method/strategy for cancer prognostic marker selection and offers valuable methodological directions to similar research topics.

## 1. Introduction

Cancer is the second leading-frequent cause of death worldwide. The global cancer burden has risen up to 18.1 million new cases and 9.6 million deaths in 2018; the incidence of cancer is expected to increase by 50% until 2040, with approximately 27 million new cases per year [1]. The American Cancer Society estimates that there will be about 1,806,590 cancer cases diagnosed and 606,520 deaths from cancer in US in 2020 [2]. New diagnostic tools and treatment guidelines have been extensively studied and developed; however, the survival outcomes of some digestive tract tumors are not showing corresponding improvement [3,4,5]. Therefore, it is still urgent to develop more reliable prognostic methods for cancer treatment guidance, especially for some digestive tract cancers, which are often asymptomatic at the early stages [6,7,8,9,10].

MicroRNAs (miRNAs) have been found to be important players in cancer development. miRNAs are a class of noncoding RNAs of about 18–22 nucleotides (nt) in length, which play key functions in the regulation of vital biological processes such as cell division and death, cellular metabolism, intracellular signaling, immunity and cell movement [11,12,13]. Rich evidence has confirmed the causal link between the dysregulation of miRNAs and cancer [14], and miRNA signatures for particular cancers have been identified by comparing tumor samples and healthy controls. Studies also suggested that the pattern of miRNA expression is associated with cancer type, stage, and other clinical variables [15]. Furthermore, the prognostic value of miRNAs has been implicated in multiple cancers including breast cancer [16], pancreatic cancer [17], hepatocellular carcinoma [18], prostate cancer [19], lung cancer [20] and others. Moreover, valuable diagnostic and prognostic biomarkers have been identified in several specific cancers by integrating datasets from different studies [21,22].

Despite extensive studies on cancer-associated miRNAs have been conducted, most attention has been paid on associations between miRNA expression aberration and individual cancer types. Evidence showed that several key miRNAs play similar and important roles in a specific groups of cancers. For example, miR-21 is associated with the survival outcomes of multiple cancers, including hepatocellular carcinoma, colon cancer and others [23,24]. Furthermore, pancancer analysis also discovered similar miRNA alterations among different cancers [25]. Therefore, we hypothesized that integrative analysis of miRNA signatures based on cancer clusters will identify more systematic and reliable biomarkers with improved prognostic power.

**Figure 1:**
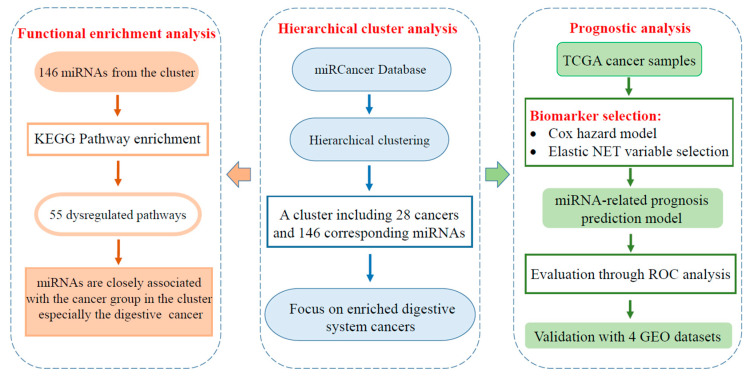
A flow chart of the study design. First, we utilized hierarchical clustering to identify clusters of cancers and associated miRNAs. In this study, we focused on the digestive system cancer enriched in the identified cluster. Furthermore, we conducted a functional enrichment analysis with cluster-associated miRNAs. Next, with data of tumor samples from The Cancer Genome Atlas (TCGA) datasets, miRNA signatures were selected for prognosis of each cancer type of six digestive system cancers. The predictive value of identified miRNA signatures was evaluated through ROC curves and validated with independent Gene Expression Omnibus (GEO) datasets.

In this study, we provide a novel cluster-based approach to identify miRNA sets for improved prediction of overall cancer survival. The detailed study design of our analysis is displayed in Figure 1. With reported aberrantly expressed miRNAs, we identified a distinct cluster including 28 cancers and associated 146 miRNAs. Digestive system cancers were enriched in the identified cluster, including stomach adenocarcinoma (STAD), esophageal carcinoma (ESCA), liver hepatocellular carcinoma (LIHC), pancreatic adenocarcinoma (PAAD), colon adenocarcinoma (COAD) and rectum adenocarcinoma (READ). In this study, we focused on these digestive system cancers and select prognostic miRNAs by analyzing RNA-seq data from The Cancer Genome Atlas (TCGA). The prognosis value of identified miRNA signatures was validated on data from independent studies.

## 2. Results

### 2.1. A Distinct Cluster of Association between miRNA Dysregulation and Cancer

**Figure 2:**
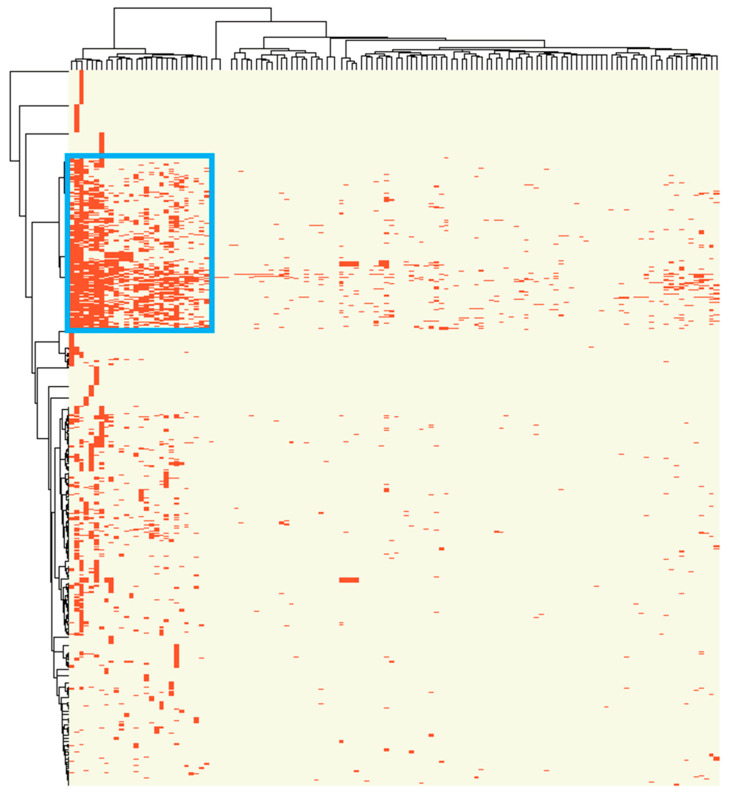
Clustering of miRNA-cancer associations. The clustering was based on the miRCancer database, with 947 miRNA and 131 tumors collected from published peer-reviewed scientific articles. The rows show miRNA and the columns show cancer types. The reported miRNA aberrations are shown in red. A significant cluster with 28 cancers and146 associated miRNAs is shown in a blue box.

With reported associations of 947 dysregulated miRNA with 131 human cancers collected in the miRCancer database, we performed hierarchical clustering and identified similar associations between cancers and miRNA dysregulation. Distinctly, a cluster including 28 cancers and 146 associated miRNAs was identified (Figure 2). Among those 28 cancers within this cluster, digestive system cancers were enriched (*n* = 8, including gastric cancer, hepatocellular carcinoma, rectum cancer, colon cancer, esophageal squamous cell carcinoma, pancreatic ductal adenocarcinoma, pancreatic cancer, esophageal cancer), followed by head and neck squamous cell carcinomas (*n* = 5), respiratory system cancers (*n* = 4), genital system cancers (*n* = 4), nervous system-related cancers (*n* = 4) and others. In this study, we focused on the cluster of digestive tract cancers in the following prognostic signature model development. The eight digestive system cancers in this cluster were mapped to six cancer types studied in TCGA, including STAD, ESCA, LIHC, PAAD, COAD and READ. The basic demographic and clinical information of patients with these interested cancers in TCGA were summarized in Supplementary Table S1.

### 2.2. Cluster Associated miRNAs Were Involved in Digestive System Related Pathways

We then investigated functions of those 146 miRNAs identified in the above distinct cluster, which may provide new implications about important regulatory mechanisms specifically for the cluster of cancers. KEGG pathway enrichment analysis identified 55 significant enriched pathways (FDR < 0.05, Supplementary Table S2). Among them, mucin type O-Glycan biosynthesis was the most significant pathway (FDR = 1.97 × 10^−12^); mucin type O-Glycan is the main component of mucins that are highly expressed on the intestinal tract and correlated with intestinal homeostasis [26] and colorectal cancer [27]. Associated with digestive system cancers, many other glycan-related pathways such as N-Glycan biosynthesis (FDR = 2.19 × 10^−4^), proteoglycans in cancer (FDR = 1.03 × 10^−6^) and other types of O-glycan biosynthesis (FDR = 1.11 × 10^−4^) were also actively involved. In addition, we found metabolism-related pathways such as fatty acid biosynthesis (FDR = 0.03) and key cancer signaling pathways such as TGF-β pathway (FDR = 2.85 × 10^−5^) were also significantly enriched. These results indicate that miRNA signatures identified in this cluster strongly link to digestive system cancers.

### 2.3. miRNA Signatures for Digestive System Cancer Prognosis

**Figure 3:**
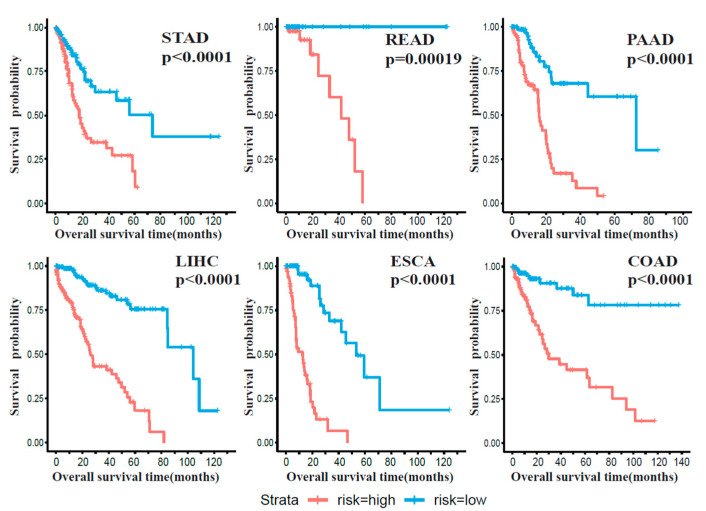
Kaplan–Meier curve of the prognostic value of miRNA signature in six digestive system cancers. Kaplan–Meier curves of high-risk and low-risk groups of patients from TCGA database in each cancer classified by prognostic models with identified miRNA signatures. All prognostic models in six cancers show significantly discriminative power between high-risk and low-risk groups.

Filtering out miRNAs with a large proportion of missing data in TCGA samples (see the Methods section), we identified 92 candidate signatures from those identified 146 cluster-associated miRNAs. Among the 92 candidate signatures, 35 in STAD, 6 in READ, 48 in LIHC, 15 in ESCA, 47 in COAD and 49 in PAAD were found to be significantly associated with survival by fitting univariate Cox proportional hazards regression. 86 miRNAs were shared by at least three tumors (Supplementary Table S3). Further, we used a regularized regression method, elastic net, to select the most important signatures in order to reduce the overfitting problem. Prognostic miRNAs were selected for each cancer type, including 8 miRNAs for STAD, 5 for READ, 24 for LIHC, 12 for ESCA, 18 for COAD and 18 for PAAD (Supplementary Tables S4–S9). For each cancer type, prognostic models based on its specific miRNA signatures were trained using a multivariate Cox proportional hazards regression, and PRS for each patient was calculated (see Methods section). Based on the median of PRS, subjects with a particular cancer type were distinctly discriminated into high-risk and low-risk groups, for all six cancers (Figure 3, STAD: *p*-value < 0.0001; READ: *p*-value = 0.00019; LIHC: *p*-value < 0.0001; COAD: *p*-value < 0.0001; ESCA: *p*-value < 0.0001; PAAD: *p*-value < 0.0001).

**Figure 4:**
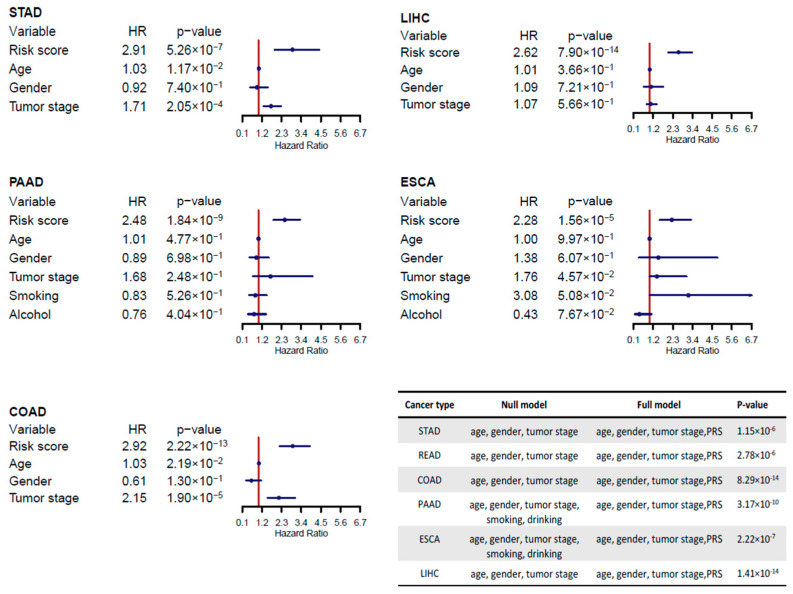
Prognostic effects of risk score and covariates. In each cancer, hazard rations (HRs) and 95% CIs estimated from Cox proportional hazards regression model were visualized in the forest plots. For categorical data of gender, smoking and drinking histories, male, smoker, and drinking were used as references respectively. Tumor stage were treated as a continuous variable in this analysis. ANOVA test was applied to compare the full model including prognostic risk scores (PRS) and covariates with the null model including covariates only. The full model showed significantly better performance in all cancers.

We also included the effects of covariates (age, gender and tumor stage) in the multivariate Cox proportional hazards regression models for all cancer types. Smoking history and alcohol history were also taken into the consideration in the analyses of PAAD and ESCA data. The estimated hazard ratios (HRs) and 95% confidence intervals (CIs) of PRS and covariates showed that PRS is the most significant prognostic factor compared with all other considered covariates (Figure 4, STAD: HR = 2.91; LIHC: HR = 2.62; PAAD: HR = 2.48; ESCA: HR = 2.28; COAD: HR = 2.92). Including PRS in the model with age, gender, tumor stage, smoking and alcohol history significantly improved the prognostic power (ANOVA test, STAD: *p*-value = 1.15 × 10^−6^, READ: *p*-value = 2.78 × 10^−6^; COAD: *p*-value = 8.29 × 10^−14^; LIHC: *p*-value = 1.41 × 10^−14^; PAAD: *p*-value = 2.58 × 10^−9^; ESCA: *p*-value = 2.42 × 10^−7^). Consistent with previous results, the covariate adjusted analyses also showed high discriminative power in Kaplan–Meier survival curves between the two groups in all six digestive system cancers (*p*-value < 0.05, Supplementary Figure S1).

### 2.4. Cluster-Based miRNA Prognostic Signatures Are Superior to Cancer-Specific Signatures

**Figure 5:**
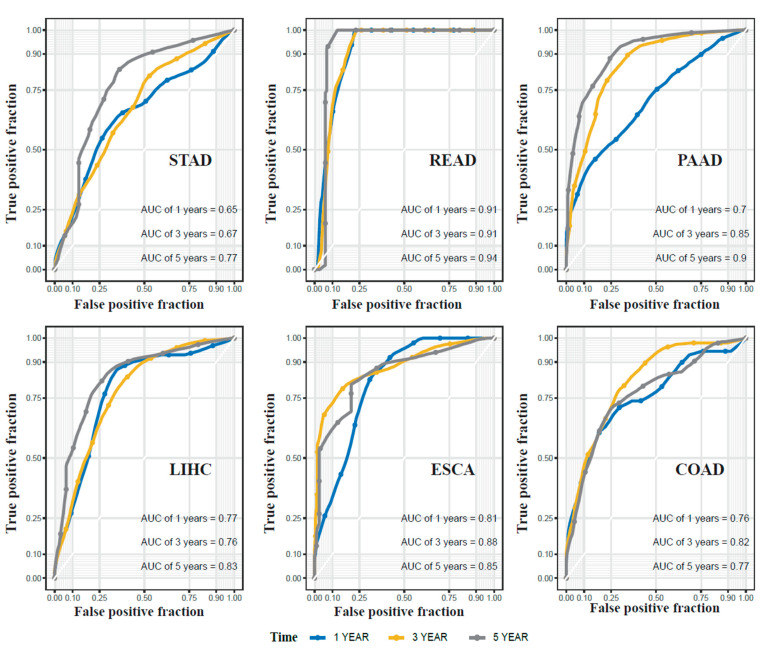
ROC curve of the prognostic value of miRNA signature in six digestive system cancers.

To demonstrate the advantage of our new method (referred to as the “cluster-based approach”) in selecting the prognostic miRNAs, we compared its prognostic ROC curves with those selected from all reported miRNAs dysregulated in specific cancers (referred to as “cancer-specific approach”). For the cancer-specific approach, 191 dysregulated miRNAs in STAD, 150 in READ, 195 in LIHC, 85 in ESCA, 71 in COAD and 64 in PAAD were identified from the miRCancer database. Similar to the cluster-based approach, univariate Cox proportional hazards regression and the elastic net variable selection model were applied, and we identified 9 prognostic miRNAs for STAD, 10 for READ, 21 for LIHC, 13 for ESCA, 19 for COAD and 16 for PAAD. Their prognostic value was also evaluated using the same method with the cluster-based approach by the Kaplan–Meier (K-M) survival curves (Supplementary Figure S2). We compared the ROC curves of survival analysis for each cancer type using cluster-based signatures (Figure 5) and cancer-specific signatures (Supplementary Figure S3). Significantly, the cluster-based prognostic model showed higher or comparable 5-year area under receiver operating characteristic curve (AUC) values than the cancer-specific prognostic model (STAD: 0.77 vs. 0.67; READ: 0.94 vs. 0.92; PAAD: 0.9 vs. 0.83; LIHC: 0.83 vs. 0.84; ESCA: 0.85 vs. 0.72; COAD: 0.77 vs. 0.74), indicating the novel miRNA signatures we selected through cluster-based approach more accurately predicted the prognosis of digestive system cancers. After adjusting the covariates including age, gender and tumor stage, cluster-based signatures consistently showed high prognostic power with 5-year AUC values as 0.73, 0.98, 0.81, 0.82, 0.78 and 0.77 in STAD, READ, PAAD, LIHC, ESCA and COAD, respectively (Supplementary Figure S4). We also selected signatures from the combination of candidate miRNA signatures of both cluster-based and cancer-specific approaches, and compared the performances of three sets of signatures (cancer-specific, cluster-based and combined) in Table 1. In LIHC and READ, we observed similarly high AUC values for all three approaches. In other four cancers, the cancer-specific signatures produced an obviously lower AUC value than signatures identified from the cluster-based approach or the combined approach.

The value of these three sets of signatures (cancer-specific, cluster-based and combined) was also assessed by cross-validation in each of five cancers (STAD, PAAD, ESCA, COAD and LIHC) (see Method). Cross-validation failed on READ due to its small number of death cases (*n* = 9). In four cancer types (STAD, PAAD, ESCA and COAD), both cluster-based and combined-approach signatures showed similar or better performance than the cancer-specific signatures on the test data (Table 1). In LIHC, we did not observe such superior performance of cluster-based miRNA signatures.

### 2.5. Validation of the Prognostic miRNA Signatures Using Independent Datasets

The prognostic value of our identified miRNA signatures for digestive system cancers were validated with multiple independent datasets, including the GSE29622 dataset for COAD, the GSE31384 dataset for LIHC, the GSE43732 dataset for ESCA and the GSE62498 dataset for PAAD. The basic demographic and clinical information of the patients in these validation datasets are summarized in Supplementary Table S10 and the validation results are shown in the Table 2. For 3 (COAD, LIHC and PAAD) out of these four datasets, the cluster-based signatures showed significant prognostic power (COAD: high-risk vs. low-risk *p* = 0.0015, 5-year-AUC = 0.81; LIHC: high-risk vs. low-risk *p* = 9.58 × 10^−6^, 5-year-AUC = 0.82; PAAD: high-risk vs. low-risk *p* = 0.0002, 5-year-AUC = 0.79), which were superior to that of cancer-specific signatures (COAD: high-risk vs. low-risk *p* = 0.0019, 5-year-AUC = 0.73; LIHC: high-risk vs. low-risk *p* = 0.0003, 5-year-AUC = 0.69; PAAD: high-risk vs. low-risk *p* = 0.2465, 5-year-AUC = 0.59). Likewise, the signatures based on the combined approach (COAD: high-risk vs. low-risk *p* = 5.87 × 10^−5^, 5-year-AUC = 0.83; LIHC: high-risk vs. low-risk *p* = 0.00014, 5-year-AUC = 0.75; PAAD: high-risk vs. low-risk *p* = 0.00003, 5-year-AUC = 0.89) demonstrated better performance than the cancer-specific signatures. No such prognostic power of the cluster-based signatures was observed in the GSE43732 ESCA dataset. Consistently, all approaches showed relatively low AUC values in ESCA. This is possibly because miRNAs in GSE43732 do not exactly match with the TCGA data with “-3p” and “-5p” miRNAs unannotated.

## 3. Discussion

In this study, we provide a new approach on improved identification of miRNA signatures for cancer prognosis and we have applied this approach on a cluster of digestive system cancers. Rather than analyzing differentially expressed miRNAs in a particular cancer type, we for the first time identified prognostic miRNA signatures based on clusters. In this study, we identified a digestive system cancers-enriched cluster including 28 cancers and 146 associated miRNAs. These miRNAs identified important pathways in digestion and metabolism were involved in this cluster of cancers.

The traditional cancer-specific approach suffers from power loss due to large heterogeneity, data noise, and bias in sampling and measurement, especially for those which have not been studied transcriptome-wide and/or those are rare with small sample sizes. Effectively, our new cluster-based approach makes greater advantage of the identified miRNA dysregulation by extensive cancer research. Borrowing information from similar cancers and miRNAs in a cluster, our approach extracts and utilizes valuable information for cancers within the interested cluster(s), leading to improved identification of key miRNAs in corresponding cancers. As shown in this study, the new cluster-based method gained significantly enhanced power of prognosis. We also identified 18 prognostic miRNAs for PAAD, including 13 miRNAs whose prognostic value were previously reported, whereas other 5 miRNAs (mir-140, mir-433, mir-217, mir-146b and mir-99b) have limited evidence reported for the link. However, these five miRNAs have been confirmed to play important roles in the prognosis of some other digestive cancers such as STAD [28,29,30,31,32], thus our approach may provide deeper thoughts on the underlying associations between different cancers. Furthermore, our approach is useful for inferring and complementing missing/hidden associations. In this study, we successfully identified prognostic miRNAs mir-340, mir-192, mir-100 and let-7 which were not curated in the database but have been proposed by recent studies [33,34,35,36].

Our study identified new miRNA signatures for the survival of STAD, READ, LIHC, ESCA, COAD and PAAD separately. Compared with the cancer specific model, most of these prognostic miRNA signatures showed better performance in cancer prognosis in both TCGA and validation GEO datasets. Still, our study has limitations. The prognostic power of our model can be limited by the miRNA information we collected from the current database. The model could be improved when more comprehensive and reliable databases are available. Furthermore, taking more clinical prognostic factors and pathological factors which are expected to be collected in future studies will improve the prognostic power. In addition, the limited sample size of current datasets such as the TCGA datasets may influence the result of our investigation, especially in READ, which has small death numbers (*n* = 9). A future large-scale and standardized study is desirable in validating the newly identified miRNA signatures. Further functional experimental study is needed to dissect the potential important roles played by these novel signatures. Despite these limitations, our study identified novel miRNA signatures with significant prognostic value for digestive system cancers and provided a new and valuable method for cancer research, which has great potential to be extended to study other types of biomarkers in many different human diseases.

## 4. Materials and Methods

All data management, statistical analyses and visualizations were accomplished using R 3.6.2.

### 4.1. miRNA-Cancer Association

In total, 947 dysregulated miRNA in 131 human cancers were identified from the miRCancer database [37], which collected cancer related miRNA signatures reported in peer-reviewed scientific articles. The clusters of miRNA-cancer associations were obtained by hierarchical clustering and visualized in heatmap using the R “*pheatmap*” package. miRNAs in the related cluster were considered as candidate prognosis biomarkers shared by cancers in this cluster.

### 4.2. miRNA and Clinical Data of Digestive System Cancers

The miRNA-seq data and clinical information were downloaded from the TCGA website (https://portal.gdc.cancer.gov). In this study we focused on six digestive system cancers, including STAD, ESCA, LIHC, PAAD, COAD and READ. Clinical variables included the overall survival, age, gender, tumor stage, smoking history and alcohol history. Independent miRNA expression datasets by microarray for validation of our prognostic models were searched in PubMed and NCBI Gene Expression Omnibus (GEO) using the key words “digestive system cancer” and “prognosis”. We initially identified 5 cohort studies with RNA-seq data for digestive system cancers we analyzed. After filtering out 1 study with relatively small sample size (*n* = 44) and no basic clinical information such as age, gender available, we finally identified 4 publicly available datasets including the GSE29622 dataset [38] for COAD, the GSE31384 dataset [39] for LIHC, the GSE43732 dataset for ESCA and the GSE62498 dataset [40] for PAAD. The quantile normalization was performed to normalize each of these microarray datasets.

### 4.3. Identification and Evaluation of Prognostic miRNAs

We analyzed TCGA miRNA expression data and clinical information to identify prognostic miRNAs from candidates obtained from above hierarchical clustering approach. Reads per kilobase per million mapped reads (RPKM) values which represent the miRNA expression levels were analyzed at the logarithmic scale with an offset of 1. For each cancer type, any miRNA whose expression data were missing in over 30% samples was removed and the missing values of the remaining miRNAs were imputed using the k nearest neighbor (kNN) method. Further, we performed univariate Cox proportional hazards regression to identify miRNAs significantly associated with the survival of each cancer type. With these pre-elected miRNAs, an elastic net variable selection using the R package “*glmnet*” was applied to select prognostic miRNA signatures. Then, we fit the selected signatures in a multivariate Cox proportional hazards model to develop the predictive model for each cancer type. The prognostic risk scores (PRS) were estimated by $\mathrm{PRS}=\sum \left(\beta *EXP_{i}\right)$, where $EXP_{i}$ was the log(RPKM + 1) value of the i-th miRNA, and β was the estimated regression coefficient for the corresponding miRNA from the multivariate Cox hazards model. According to PRS, study samples were then categorized into a high-risk group and a low-risk group by the median of PRS as the cutoff value. The difference of the overall survival between the high-risk and low-risk groups were evaluated by Kaplan–Meier (K-M) survival curves and log-rank test. The R package “survival” was used to perform the survival analysis and *p*-values < 0.05 were considered to be statistically significant. We also evaluated the prognostic performance of the miRNA signature model by comparing the area under receiver operating characteristic curve (AUC), using R package “survival ROC”.

### 4.4. Comparison between the Cluster-Based Approach and the Cancer-Specific Approach

We refer to aforementioned approach to identify cancer prognostic markers as the cluster-based approach. Separately, we developed a cancer-specific approach for analysis with each single cancer type. For the cancer-specific approach, cancer associated miRNAs were considered as candidate prognostic miRNA for each individual cancer type. Next, prognostic miRNA biomarkers were identified using the same method as that used in the cluster-based approach. ROC curve was also used to evaluate the prognosis value of miRNA signatures identified by the cancer-specific approach. Using the same strategy, we also combined all candidate prognostic miRNA signatures from the cluster-based and cancer-specific approaches, identified prognostic signatures, and evaluated their combined prognostic value. The AUC values of 1-year, 3-year and 5-year from these three approaches (cluster-specific, cancer-specific and combined) were compared.

### 4.5. Validation of Prognostic miRNAs

To validate the prognosis performance of selected signatures for each cancer type, a cross-validation strategy was used. All samples were randomly split into a training dataset (70% of all samples) and a test dataset (30% of all samples). The prognosis model was fit on the training dataset and then the performance was assessed on the test dataset. With repeats of 100 times, the AUC values of training and test sets were calculated and averaged to evaluate the prognostic value.

The final prognostic miRNA signatures identified through the cluster-based approach, the cancer specific approach and the combined approach were further validated in four independent datasets, including GSE29622 for COAD, GSE31384 for LIHC, GSE43732 for ESCA and GSE62498 for PAAD. For each independent validation dataset, multivariate Cox hazards model was fit with prognostic miRNA signatures and PRS was calculated using the estimated coefficients. Further, AUC values were calculated to evaluate the prognostic value of these miRNA signatures in these four independent datasets, respectively.

### 4.6. Pathway Enrichment Analysis

We used DIANA-TarBase v7.0 and DIANA-miRPath v3.0 [41] to further identify functions and signal pathways involved by miRNAs associated with the interested cluster of cancers. The enrichment of Kyoto Encyclopedia of Genes and Genomes (KEGG) pathways was assessed. A false discovery rate (FDR) <0.05 was considered statistically significant.

## 5. Conclusions

In conclusion, our study identified significant miRNA signatures with improved prognosis accuracy in digestive system cancers through a novel cluster-based approach, which can integrate miRNA information identified by extensive cancer research for developing improved prognostic models and related research topics. Besides taking advantage of information from similar cancers and miRNAs in a cluster, our approach can provide valuable insights on cancer with limited studies.

## Figures and Tables

**Table 1:** Area under receiver operating characteristic curve (AUC) values of miRNA signatures models in full datasets and cross-validation. With full dataset as training dataset, cancer specific signatures showed lowest AUC values in all six cancers. The average AUC values of test sets with repeats of 100 times were calculated for cross-validation. The highest AUC and close ones (less than 0.03 in difference) are shown in bold.

Cancer	Method	Full Dataset		Cross-Validation	
		AUC-3	AUC-5	AUC-3	AUC-5
STAD	cluster-based	0.67	**0.77**	0.62	**0.67**
	cancer-specific	0.61	0.67	0.57	0.6
	combined	**0.73**	0.72	**0.67**	**0.66**
PAAD	cluster-based	**0.85**	**0.9**	**0.72**	**0.74**
	cancer-specific	0.82	0.83	0.67	0.64
	combined	**0.86**	**0.93**	**0.69**	**0.72**
ESCA	cluster-based	**0.88**	**0.85**	**0.74**	**0.7**
	cancer-specific	0.78	0.72	0.68	0.61
	combined	**0.86**	0.81	**0.73**	**0.68**
COAD	cluster-based	**0.82**	**0.77**	**0.69**	**0.61**
	cancer-specific	0.77	0.74	0.6	0.55
	combined	**0.82**	**0.79**	**0.67**	**0.61**
LIHC	cluster-based	**0.76**	**0.83**	0.65	0.68
	cancer-specific	**0.78**	**0.84**	**0.7**	**0.76**
	combined	**0.78**	**0.84**	**0.7**	**0.76**
READ	cluster-based	**0.91**	**0.94**	**-**	**-**
	cancer-specific	**0.9**	**0.92**	**-**	**-**
	combined	**0.92**	**0.91**	**-**	**-**

AUC-3: The 3-year AUC value; AUC-5: The 5-year AUC value.

**Table 2:** Validation of novel miRNA signatures in COAD, LIHC, PAAD and ESCA with GSE29622, GSE31384, GSE62498 and GSE43732. With multivariate Cox proportional hazards model, we evaluated the discrimination power of newly identified miRNA signatures between the high-risk and low-risk groups. The HR and *p*-value were shown. AUC was also used to evaluate and compare the prognostic value of miRNA signatures identified from three approaches. The highest AUC and close ones (less than 0.03 in difference) are shown in bold.

Cancer	Method	HR (95% CI)	*p*-Value	AUC-3	AUC-5
COAD	cluster-based	0.14 (0.04–0.47)	0.0015 *	0.76	**0.81**
	cancer-specific	0.18 (0.06–0.52)	0.0019 *	0.77	0.73
	combined	0.11 (0.04–0.32)	5.87 × 10^−5^ *	**0.81**	**0.83**
LIHC	cluster-based	0.22 (0.11–0.43)	9.58 × 10^−6^ *	**0.82**	**0.82**
	cancer-specific	0.39 (0.23–0.65)	0.0003 *	0.66	0.69
	combined	0.39 (0.24–0.63)	0.00014 *	0.73	0.75
PAAD	cluster-based	0.31 (0.17–0.58)	0.0002 *	0.74	0.79
	cancer-specific	0.65 (0.32–1.34)	0.2465	0.59	0.59
	combined	0.32 (0.18–0.59)	0.00003 *	**0.81**	**0.89**
ESCA	cluster-based	0.67 (0.42–1.06)	0.0871	0.57	0.58
	cancer-specific	0.49 (0.31–0.79)	0.0034 *	**0.65**	**0.65**
	combined	0.64 (0.40–1.01)	0.0583	0.61	**0.62**

* *p*-value < 0.05 was considered to be statistically significant. AUC-3: the 3-year AUC value; AUC-5: the 5-year AUC value.

## Data Availability

The data that support the findings of this study are available in miRCancer at http://mircancer.ecu.edu/ and TCGA at https://portal.gdc.cancer.gov, reference number TCGA-STAD, TCGA-ESCA, TCGA-COAD, TCGA-READ, TCGA-LIHC and TCGA-PAAD, and GEO datasets GSE29622, GSE31384, GSE43732 and GSE62498.

## Supplementary Materials

The following are available online at https://www.mdpi.com/1422-0067/22/4/1529/s1. Table S1: Demographic and clinical information of patients with digestive system cancer in the TCGA dataset. Table S2: KEGG pathway enriched by 146 miRNAs from the distinct cluster. Table S3: Candidates signatures shared at least 50% of tumors analyzed. Table S4: Univariate and multivariate Cox proportional hazard regression analyses of miRNAs in STAD. Table S5: Univariate and multivariate Cox proportional hazard regression analyses of miRNAs in PAAD. Table S6: Univariate and multivariate Cox proportional hazard regression analyses of miRNAs in READ. Table S7: Univariate and multivariate Cox proportional hazard regression analyses of miRNAs in LIHC. Table S8: Univariate and multivariate Cox proportional hazard regression analyses of miRNAs in ESCA. Table S9: Univariate and multivariate Cox proportional hazard regression analyses of miRNAs in COAD. Table S10: Demographic and clinical information of patients with digestive system cancers in independent validation datasets from GEO. Figure S1: Kaplan–Meier curves of high-risk and low-risk groups of patients from TCGA database in each cancer after adjusting age, gender, stage of cancer. Figure S2: Kaplan–Meier curves of high-risk and low-risk groups of patients classified by the cancer-specific approach from TCGA datasets in six digestive system cancers. Figure S3: ROC curve of the prognostic value of miRNA signature identified by the cancer-specific approach in six digestive system cancers. Figure S4: ROC curve of the prognostic value of miRNA signature in six digestive system cancers after adjusting age, gender and tumor stage.
