# Supplementary material for: A Cluster-Based Approach for Identifying Prognostic microRNA Signatures in Digestive System Cancers
Source: Int J Mol Sci. 2021 Feb 3;22(4):1529. doi: 10.3390/ijms22041529 (PMC7913556; doi:10.3390/ijms22041529)
Supplement: Supplementary file 1 [file ijms-22-01529-s001.pdf]

Supplementary materials

A cluster-based approach for identifying prognostic microRNA signatures in digestive system cancers

Jun Zhou[1], Xiang Cui[1], Feifei Xiao[2], Guoshuai Cai[1*]

[1]Department of Environmental Health Sciences, Arnold School of Public Health, University of South Carolina, Columbia, SC, 29208, USA

[2]Department of Epidemiology and Biostatistics, Arnold School of Public Health, University of South Carolina, Columbia, SC, 29208, USA

*Address correspondence to:

Guoshuai Cai

Department of Environmental Health Sciences

Arnold School of Public Health

University of South Carolina

915 Greene Street

Discovery 517

Columbia, SC 29204

Email: GCAI@mailbox.sc.edu

Phone: 803-777-4120

Table S1. Demographic and clinic information of patients with digestive system cancer in the TCGA dataset. The study involved 428 samples with STAD, 160 with READ, 370 with LIHC, 440 with COAD, 183 with ESCA, 178 with PAAD. Age, gender, and pathologic stage were adjusted in survival analysis. Smoking history and alcohol history were additionally taken into consideration in PAAD and ESCA analyses.

**Characteristic**	STAD	READ	LIHC	COAD	ESCA	PAAD
**N**	428	160	370	440	183	178
**Age, mean±SD**	65.67±10.74	64.28±11.85	59.50±13.37	66.78±13.15	62.55±11.91	64.58 (10.94)
**Gender, n (%)**						
Male	275 (64.3)	86 (53.8)	252 (68.1)	229 (52.0)	156 (85.2)	98 (55.1)
Female	153 (35.7)	74 (46.2)	118 (31.9)	211 (48.0)	27 (14.8)	80 (44.9)
**Vital Status, n (%)**						
alive	346 (80.8)	151 (94.4)	282 (76.2)	385 (87.5)	126 (68.9)	119 (66.9)
dead	82 (19.2)	9 (5.6)	88 (23.8)	55 (12.5)	57 (31.1)	59 (33.1)
**Survival time,mean(SD)**	9.45 (16.34)	9.15 (19.21)	20.11(23.22)	12.22(23.49)	11.71(16.67)	13.37 (15.75)
**Pathologic stage, n (%)**						
I	54 (12.6)	29 (18.1)	172 (46.5)	73 (16.6)	18 (9.8)	21 (11.8)
II	127 (29.7)	48 (30.0)	85 (23.0)	166 (37.7)	77 (42.1)	147 (82.6)
III	178 (41.6)	50 (31.2)	84 (22.7)	125 (28.4)	56 (30.6)	3 (1.7)
IV	42 (9.8)	24 (15.0)	5 (1.4)	65 (14.8)	9 (4.9)	4 (2.2)
NA	27 (6.3)	9 (5.6)	24 (6.5)	11 (2.5)	23 (12.6)	3 (1.7)
**Smoking history, n (%)**						
Non-smoker	--	--	--	--	55 (30.1)	66 (37.1)
Ever-smoker	--	--	--	--	109 (59.6)	79 (44.4)
NA	--	--	--	--	19 (10.4)	33 (18.5)
**Alcohol history, n (%)**						
NO	--	--	--	--	52 (28.4)	64 (36.0)
YES	--	--	--	--	128 (69.9)	102 (57.3)
NA	--	--	--	--	3 (1.6)	12 (6.7)

Table S2: KEGG pathway enriched by 146 miRNAs from the distinct cluster. 55 significant enriched pathways were identified. Mucin type O-Glycan biosynthesis was the most significantly enriched. Besides, many glycan-related pathways and cancer related signaling pathways were identified.

KEGG pathway	FDR	genes #	miRNAs #
Mucin type O-Glycan biosynthesis	1.97E-12	29	116
Axon guidance	6.28E-07	117	190
ECM-receptor interaction	1.03E-06	74	163
Proteoglycans in cancer	1.03E-06	176	206
ErbB signaling pathway	1.33E-05	82	193
Lysine degradation	1.61E-05	48	170
Focal adhesion	1.77E-05	186	211
TGF-beta signaling pathway	2.85E-05	74	185
Other types of O-glycan biosynthesis	1.11E-04	29	121
Hippo signaling pathway	1.11E-04	135	200
Pathways in cancer	1.11E-04	342	215
Glycosaminoglycan biosynthesis - keratan sulfate	2.19E-04	15	69
N-Glycan biosynthesis	2.19E-04	43	144
Ras signaling pathway	2.19E-04	196	214
Protein processing in endoplasmic reticulum	2.82E-04	146	198
Bacterial invasion of epithelial cells	3.12E-04	71	176
Adherens junction	3.12E-04	68	182
Pancreatic cancer	7.94E-04	61	178
Phosphatidylinositol signaling system	7.94E-04	70	180
Rap1 signaling pathway	8.57E-04	183	212
AMPK signaling pathway	8.62E-04	110	200
Renal cell carcinoma	1.17E-03	61	186
Colorectal cancer	1.25E-03	58	178

Endocytosis	1.47E-03	180	202
Neurotrophin signaling pathway	1.62E-03	108	203
Thyroid hormone signaling pathway	1.90E-03	105	200
Acute myeloid leukemia	2.03E-03	53	180
Chronic myeloid leukemia	2.92E-03	66	185
PI3K-Akt signaling pathway	5.13E-03	288	216
Regulation of actin cytoskeleton	6.04E-03	182	206
Endometrial cancer	7.31E-03	48	180
Inflammatory mediator regulation of TRP channels	7.31E-03	86	182
Glioma	7.31E-03	57	192
Platelet activation	7.88E-03	112	203
p53 signaling pathway	8.63E-03	62	171
Notch signaling pathway	8.67E-03	45	132
Choline metabolism in cancer	8.69E-03	89	203
Arrhythmogenic right ventricular cardiomyopathy (ARVC)	9.85E-03	66	167
MAPK signaling pathway	1.13E-02	215	210
Oxytocin signaling pathway	1.55E-02	135	203
Small cell lung cancer	1.66E-02	76	184
Wnt signaling pathway	1.66E-02	121	201
Signaling pathways regulating pluripotency of stem cells	1.69E-02	121	207
Long-term depression	2.30E-02	51	184
Prolactin signaling pathway	2.30E-02	61	188
Glutamatergic synapse	2.30E-02	98	199
Melanoma	2.90E-02	63	182
Dorso-ventral axis formation	3.05E-02	26	152
Fatty acid biosynthesis	3.07E-02	10	72

Type II diabetes mellitus	3.07E-02	44	161
Non-small cell lung cancer	3.31E-02	49	176
Fc gamma R-mediated phagocytosis	3.39E-02	79	182
Gap junction	3.73E-02	75	192
FoxO signaling pathway	3.86E-02	112	194
mTOR signaling pathway	4.50E-02	54	189

Table S3. Signature candidates shared at least 50% of tumors analyzed. We identified 92 candidate signatures from those identified 146 cluster-associated miRNAs and the overlapped miRNAs share by at least three tumors are listed below.

miRNA	Freq	miRNA	Freq	miRNA	Freq
hsa-mir-141	6	hsa-let-7e	4	hsa-let-7c	3
hsa-mir-145	6	hsa-mir-125a	4	hsa-let-7d	3
hsa-mir-15a	6	hsa-mir-1271	4	hsa-let-7f-1	3
hsa-mir-195	6	hsa-mir-132	4	hsa-let-7f-2	3
hsa-mir-21	6	hsa-mir-140	4	hsa-let-7g	3
hsa-mir-214	6	hsa-mir-142	4	hsa-let-7i	3
hsa-mir-34a	6	hsa-mir-146b	4	hsa-mir-106b	3
hsa-mir-375	6	hsa-mir-148a	4	hsa-mir-130b	3
hsa-mir-100	5	hsa-mir-148b	4	hsa-mir-139	3
hsa-mir-10b	5	hsa-mir-152	4	hsa-mir-197	3
hsa-mir-126	5	hsa-mir-17	4	hsa-mir-19a	3
hsa-mir-143	5	hsa-mir-182	4	hsa-mir-20a	3
hsa-mir-146a	5	hsa-mir-191	4	hsa-mir-217	3
hsa-mir-150	5	hsa-mir-192	4	hsa-mir-222	3
hsa-mir-155	5	hsa-mir-200b	4	hsa-mir-23b	3
hsa-mir-15b	5	hsa-mir-200c	4	hsa-mir-26b	3
hsa-mir-183	5	hsa-mir-210	4	hsa-mir-32	3
hsa-mir-18a	5	hsa-mir-212	4	hsa-mir-326	3
hsa-mir-200a	5	hsa-mir-22	4	hsa-mir-338	3
hsa-mir-204	5	hsa-mir-224	4	hsa-mir-33a	3
hsa-mir-221	5	hsa-mir-25	4	hsa-mir-340	3
hsa-mir-223	5	hsa-mir-30a	4	hsa-mir-495	3
hsa-mir-27a	5	hsa-mir-335	4	hsa-mir-98	3

hsa-mir-29a	5	hsa-mir-429	4	hsa-mir-99a	3
hsa-mir-29c	5	hsa-mir-433	4	hsa-mir-106a	2
hsa-mir-31	5	hsa-mir-497	4	hsa-mir-144	2
hsa-mir-34c	5	hsa-mir-590	4	hsa-mir-186	2
hsa-mir-494	5	hsa-mir-96	4	hsa-mir-370	2
hsa-mir-503	5	hsa-let-7a-1	3	hsa-mir-940	2
hsa-mir-93	5	hsa-let-7a-2	3	hsa-mir-99b	2
hsa-let-7b	4	hsa-let-7a-3	3		

Table S4: Univariate and multivariate Cox proportional hazard regression analyses of miRNAs in STAD. In univariate Cox proportional hazard regression analyses, *p*-values < 0.5 were considered to be statistically significant.

	miRNA	Univariate		Multivariate	
		HR (95% CI)	P value	HR (95% CI)	P value
STAD (8 markers)	hsa-let-7d	0.76 (0.62-0.94)	9.91E-03	0.79(0.62-1.02)	6.73E-02
	hsa-mir-100	1.2 (0.92-1.5)	2.12E-01	1.27(0.98-1.65)	6.53E-02
	hsa-mir-106b	0.8 (0.66-0.96)	1.83E-02	0.84(0.68-1.03)	1.00E-01
	hsa-mir-200c	0.85 (0.7-1)	1.10E-01	0.81(0.66-1.01)	6.17E-02
	hsa-mir-20a	1.2 (0.87-1.5)	3.04E-01	1.33(0.98-1.82)	7.12E-02
	hsa-mir-29a	1.2 (0.92-1.4)	2.17E-01	1.14(0.88-1.47)	3.22E-01
	hsa-mir-370	0.8 (0.64-0.99)	3.95E-02	0.9(0.71-1.15)	3.97E-01
	hsa-mir-96	0.79 (0.64-0.98)	3.58E-02	0.74(0.58-0.94)	1.55E-02

Table S5: Univariate and multivariate Cox proportional hazard regression analyses of miRNAs in PAAD. In univariate Cox proportional hazard regression analyses, p-values < 0.5 were considered to be statistically significant.

	miRNA	Univariate*		Multivariate	
		HR (95% CI)	P value	HR (95% CI)	P value
PAAD (18 markers)	hsa-let-7d	0.63 (0.49-0.82)	4.24E-04	1.14(0.67-1.94)	6.20E-01
	hsa-let-7e	1.1 (0.83-1.5)	4.63E-01	1.4(0.95-2.05)	8.52E-02
	hsa-mir-100	1.1 (0.85-1.5)	3.90E-01	1.42(0.99-2.05)	5.99E-02
	hsa-mir-140	1.1 (0.82-1.5)	4.50E-01	1.1(0.73-1.64)	6.48E-01
	hsa-mir-143	1.3 (0.97-1.8)	7.61E-02	1.81(1.19-2.74)	5.48E-03
	hsa-mir-145	0.86 (0.67-1.1)	2.10E-01	0.63(0.43-0.92)	1.72E-02
	hsa-mir-146b	0.83 (0.63-1.1)	1.91E-01	0.79(0.56-1.1)	1.65E-01
	hsa-mir-152	0.78 (0.59-1)	9.66E-02	0.75(0.53-1.08)	1.26E-01
	hsa-mir-186	0.66 (0.52-0.85)	1.21E-03	1.17(0.83-1.65)	3.69E-01
	hsa-mir-191	0.66 (0.52-0.85)	1.38E-03	0.72(0.44-1.18)	1.95E-01
	hsa-mir-192	0.77 (0.6-0.98)	3.12E-02	0.71(0.5-1.03)	6.94E-02

hsa-mir-21	1.7 (1.2-2.4)	6.02E-03	1.36(0.83-2.22)	2.19E-01
hsa-mir-212	1.2 (0.86-1.6)	3.11E-01	1.63(1.04-2.54)	3.23E-02
hsa-mir-217	0.65 (0.51-0.83)	6.36E-04	0.72(0.52-1.02)	6.32E-02
hsa-mir-335	0.71 (0.54-0.92)	9.89E-03	0.63(0.44-0.89)	9.87E-03
hsa-mir-340	0.67 (0.51-0.89)	5.43E-03	0.9(0.63-1.27)	5.41E-01
hsa-mir-433	0.81 (0.63-1)	1.08E-01	0.81(0.56-1.18)	2.77E-01
hsa-mir-99b	0.66 (0.53-0.83)	4.39E-04	0.81(0.55-1.17)	2.59E-01

Table S6: Univariate and multivariate Cox proportional hazard regression analyses of miRNAs in READ. Considering the limited death case of READ in TCGA, *p*-values < 0.05 were considered to be statistically significant in univariate Cox proportional hazard regression analyses.

	miRNA	Univariate*		Multivariate	
		HR (95% CI)	P value	HR (95% CI)	P value
READ (5 markers)	hsa-let-7d	11 (1.6-78)	1.49E-02	4.62(0.28-76.55)	2.85E-01
	hsa-let-7f-2	0.39 (0.16-0.91)	2.96E-02	0.35(0.12-1.03)	5.74E-02
	hsa-let-7g	0.37 (0.17-0.81)	1.25E-02	0.56(0.21-1.55)	2.67E-01
	hsa-mir-21	0.13 (0.033-0.53)	4.11E-03	0.09(0.01-1.02)	5.21E-02
	hsa-mir-210	0.53 (0.3-0.93)	2.69E-02	0.51(0.22-1.19)	1.21E-01

Table S7: Univariate and multivariate Cox proportional hazard regression analyses of miRNAs in LIHC. In univariate Cox proportional hazard regression analyses, *p*-values < 0.5 were considered to be statistically significant.

	miRNA	Univariate		Multivariate	
		HR (95% CI)	P value	HR (95% CI)	P value
LIHC (24 markers)	hsa-let-7f-1	0.84 (0.69-1)	5.72E-02	0.8(0.66-0.99)	3.71E-02
	hsa-mir-100	0.85 (0.7-1)	9.68E-02	0.96(0.77-1.19)	7.03E-01
	hsa-mir-143	0.87 (0.72-1)	1.45E-01	0.96(0.78-1.2)	7.38E-01
	hsa-mir-144	1.4 (1-1.9)	2.59E-02	1.49(1.06-2.11)	2.25E-02
	hsa-mir-146a	0.8 (0.63-1)	5.51E-02	0.75(0.57-0.97)	2.69E-02
	hsa-mir-148a	1.2 (0.92-1.5)	1.77E-01	1.11(0.83-1.48)	4.98E-01
	hsa-mir-152	1.3 (1-1.7)	3.45E-02	1.22(0.94-1.59)	1.43E-01
	hsa-mir-18a	0.86 (0.7-1.1)	1.53E-01	0.86(0.67-1.09)	2.11E-01
	hsa-mir-200a	1.2 (0.91-1.5)	2.05E-01	1.23(0.92-1.64)	1.55E-01
	hsa-mir-204	0.75(0.62-0.91)	2.91E-03	0.79(0.63-1)	5.27E-02
	hsa-mir-21	1.2 (0.94-1.5)	1.37E-01	1.13(0.87-1.48)	3.57E-01
	hsa-mir-222	1.3 (0.98-1.8)	6.49E-02	1.16(0.83-1.63)	3.75E-01
	hsa-mir-25	1.3 (1-1.8)	4.67E-02	1.35(0.99-1.85)	5.92E-02

hsa-mir-26b	1.2 (0.92-1.5)	1.74E-01	1.06(0.78-1.43)	7.14E-01
hsa-mir-29a	0.73 (0.59-0.9)	2.95E-03	0.83(0.65-1.05)	1.27E-01
hsa-mir-29c	0.88 (0.71-1.1)	2.46E-01	0.91(0.72-1.15)	4.23E-01
hsa-mir-30a	1.3 (0.98-1.6)	6.64E-02	1.27(0.96-1.68)	9.64E-02
hsa-mir-335	1.2 (0.96-1.5)	1.14E-01	1.11(0.88-1.39)	3.90E-01
hsa-mir-34a	0.87 (0.71-1.1)	1.93E-01	0.88(0.7-1.1)	2.66E-01
hsa-mir-34c	1.2 (0.93-1.5)	1.78E-01	1.18(0.89-1.58)	2.55E-01
hsa-mir-370	1.4 (1-1.9)	3.30E-02	1.29(0.92-1.8)	1.35E-01
hsa-mir-940	1.2 (0.95-1.6)	1.23E-01	1.19(0.88-1.62)	2.64E-01
hsa-mir-98	0.87 (0.7-1.1)	1.77E-01	0.84(0.65-1.08)	1.73E-01
hsa-mir-99b	0.92 (0.77-1.1)	3.54E-01	0.85(0.68-1.07)	1.67E-01

Table S8: Univariate and multivariate Cox proportional hazard regression analyses of miRNAs in ESCA. In univariate Cox proportional hazard regression analyses, *p*-values < 0.1 were considered to be statistically significant.

	miRNA	Univariate		Multivariate	
		HR (95% CI)	P value	HR (95% CI)	P value
ESCA (12 markers)	hsa-let-7a-2	1.5 (1.1-2.2)	1.81E-02	1.61(1.03-2.51)	3.60E-02
	hsa-let-7i	1.5 (1.1-2.1)	2.05E-02	1.97(1.24-3.12)	4.07E-03
	hsa-mir-106a	1.3 (0.96-1.9)	8.63E-02	1.21(0.84-1.74)	3.01E-01
	hsa-mir-126	1.4 (0.96-1.9)	8.84E-02	1.38(0.9-2.1)	1.37E-01
	hsa-mir-155	1.3 (0.96-1.7)	8.93E-02	1.13(0.81-1.59)	4.68E-01
	hsa-mir-17	0.77 (0.59-1)	4.84E-02	0.81(0.59-1.11)	1.86E-01
	hsa-mir-186	1.5 (1-2.2)	4.90E-02	1.39(0.91-2.14)	1.30E-01
	hsa-mir-18a	1.5 (1.1-2.1)	2.48E-02	1.33(0.87-2.05)	1.92E-01
	hsa-mir-223	0.7 (0.53-0.92)	1.00E-02	0.73(0.55-0.97)	3.28E-02
	hsa-mir-23b	1.5 (1.1-2)	2.25E-02	1.49(1.02-2.17)	3.80E-02
	hsa-mir-29a	1.4 (1.1-1.9)	1.51E-02	1.29(0.94-1.78)	1.14E-01
	hsa-mir-370	0.8 (0.62-1)	8.40E-02	0.75(0.56-0.99)	4.56E-02

Table S9: Univariate and multivariate Cox proportional hazard regression analyses of miRNAs in COAD. In univariate Cox proportional hazard regression analyses, *p*-values < 0.5 were considered to be statistically significant.

	miRNA	Univariate		Multivariate	
		HR (95% CI)	P value	HR (95% CI)	P value
COAD (18 markers)	hsa-let-7c	1.3 (0.94-1.8)	1.15E-01	1.24(0.81-1.89)	3.19E-01
	hsa-let-7e	0.9 (0.72-1.1)	3.51E-01	0.74(0.55-1.01)	5.65E-02
	hsa-let-7g	1.2 (0.95-1.6)	1.17E-01	1.15(0.85-1.54)	3.59E-01
	hsa-mir-132	1.3 (0.95-1.7)	9.80E-02	1.23(0.89-1.69)	2.05E-01
	hsa-mir-142	1.2 (0.95-1.6)	1.15E-01	1.16(0.85-1.6)	3.47E-01
	hsa-mir-143	0.79 (0.6-1)	9.93E-02	0.68(0.48-0.96)	3.03E-02
	hsa-mir-148a	0.86 (0.68-1.1)	2.12E-01	0.77(0.54-1.1)	1.48E-01

hsa-mir-17	1.5 (1-2.1)	3.83E-02	1.06(0.73-1.56)	7.53E-01
hsa-mir-183	1.3 (0.92-1.8)	1.38E-01	1.84(1.09-3.1)	2.31E-02
hsa-mir-18a	0.91 (0.72-1.2)	4.56E-01	0.72(0.52-1)	5.15E-02
hsa-mir-200a	0.81 (0.67-1)	4.58E-02	0.66(0.48-0.9)	7.95E-03
hsa-mir-204	1.7 (1.1-2.8)	2.33E-02	1.26(0.73-2.16)	4.06E-01
hsa-mir-21	0.81 (0.62-1)	1.03E-01	0.66(0.49-0.89)	7.17E-03
hsa-mir-26b	0.91 (0.74-1.1)	3.98E-01	0.75(0.56-1.01)	5.89E-02
hsa-mir-32	1.5 (1-2.1)	4.76E-02	2.08(1.22-3.54)	7.10E-03
hsa-mir-433	1.5 (1-2.1)	3.29E-02	1.51(0.99-2.3)	5.67E-02
hsa-mir-93	0.91 (0.73-1.1)	3.70E-01	0.72(0.52-0.99)	4.55E-02
hsa-mir-98	0.88 (0.71-1.1)	2.39E-01	0.88(0.65-1.2)	4.35E-01

Table S10. Demographic and clinic information of patients with digestive system cancers in independent validation datasets from GEO. The study involved 65 samples with COAD, 166 with LIHC, 119 with ESCA, 65 with PAAD.

**Characteristic**	GSE29622	GSE31384	GSE43732	GSE62498
**N**	65	166	119	65
**Cancer type**	COAD	LIHC	ESCA	PAAD
**Age, mean±SD**	-	-	59.03 ± 9	-
**Gender, n (%)**				
Male	40(61.5%)	-	98(82.4%)	-
Female	25(38.5%)	-	21(17.6%)	-
**Vital Status, n (%)**				
alive	40(61.5%)	93(56%)	46(38.7%)	16(23.2%)
dead	25(38.5%)	73(44%)	73(61.3%)	49(71%)
**Survival time ,mean(SD)**	45.87(29)	32.01(20)	37.06(24)	20.4(17)
**Pathologic stage,n (%)**				
I	7(10.8%)	-	6(5%)	-
II	22(33.8%)	-	47(39.5%)	-
III	18(27.7%)	-	66(55.5%)	-
IV	18(27.7%)	-	0(0%)	-

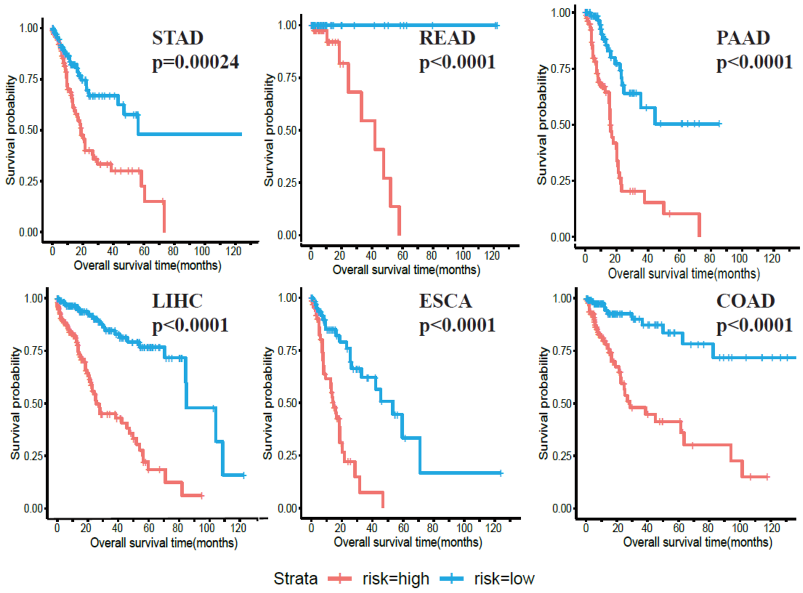

Figure S1: **Kaplan-Meier curves of high-risk and low-risk groups of patients from TCGA database in each cancer after adjusting age, gender, stage of cancer**. Smoking history and alcohol history were additionally taken into the consideration in PAAD and ESCA analyses. All the models showed significantly discriminative power in Kaplan-Meier survival curves between high-risk and low-risk groups

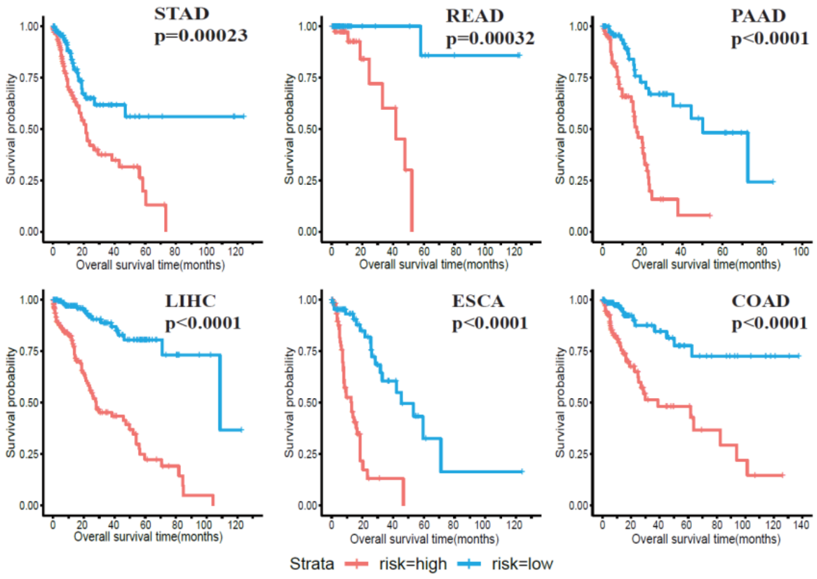

Figure S2: Kaplan-Meier curves of high-risk and low-risk groups of patients classified by the cancer-specific approach from TCGA datasets in six digestive system cancers. All the models showed significantly discriminative power in high-risk and low-risk groups.

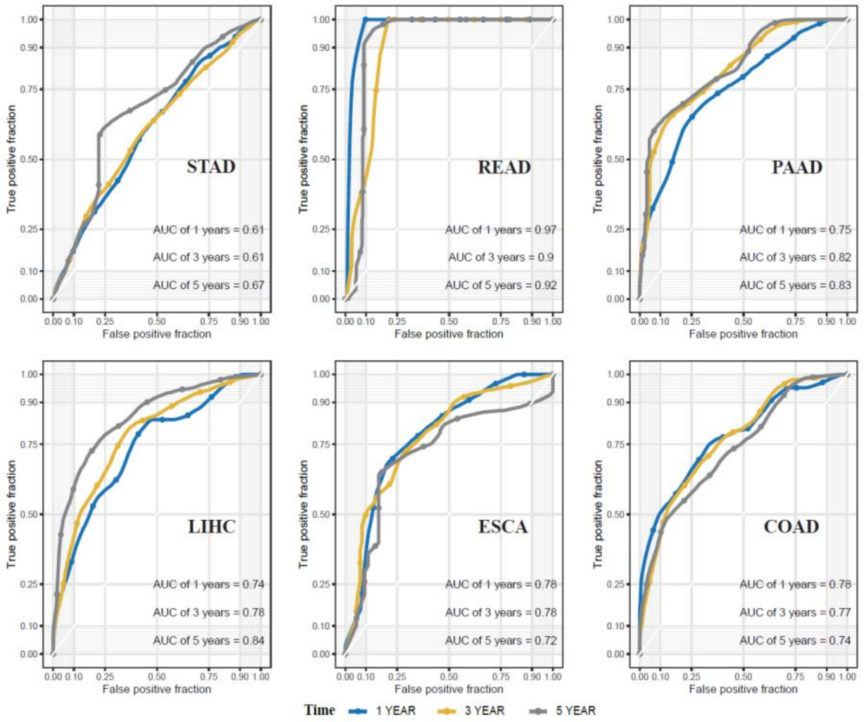

Figure S3: ROC curve of the prognostic value of miRNA signature identified by the cancer-specific approach in six digestive system cancers.

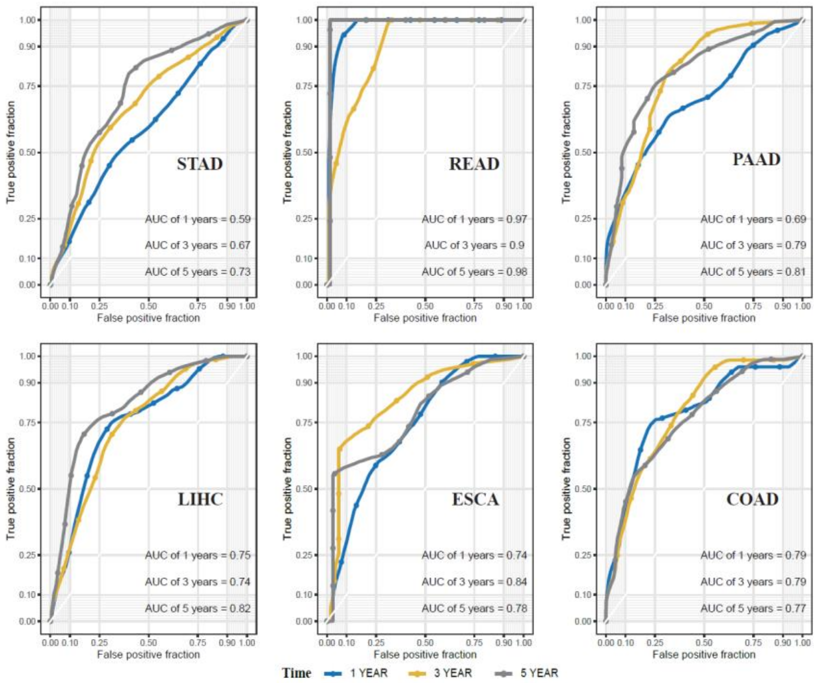

Figure S4: ROC curve of the prognostic value of miRNA signature in six digestive system cancers after adjusting age, gender and tumor stage.
